# Comparative expression of cyclooxygenase 2 and Ki67 in amelanotic and conventional oral melanomas

**DOI:** 10.4317/medoral.23737

**Published:** 2020-05-10

**Authors:** Ciro Dantas Soares, Juan Carlos Hernandez-Guerrero, Bruno Augusto Benevenuto de Andrade, Mário José Romañach, Adalberto Mosqueda-Taylor, Román Carlos, Madja Ruanna Soares Macedo, Oslei Paes de Almeida, Jacks Jorge

**Affiliations:** 1 Department of Oral Diagnosis, Area of Pathology, Piracicaba Dental School, University of Campinas, Piracicaba, São Paulo, Brazil; 2 Immunology Laboratory, Faculty of Dentistry, National University Autonomous of Mexico; 3 Oral Pathology, School of Dentistry, Federal University of Rio de Janeiro (UFRJ), Rio de Janeiro, Brazil; 4 Health Care Department, Universidad Autónoma Metropolitana, Xochimilco, Mexico; 5 Pathology Division, Centro Clínico de Cabeza y Cuello/Hospital Herrera Llerandi, Guatemala City, Guatemala; 6 Department of Nursing, Federal University of Rio Grande do Norte, Natal, Rio Grande do Norte, Brazil

## Abstract

**Background:**

Oral melanomas have some histopathological resemblance with its cutaneous counterpart; however, an aggressive behavior is more common in tumors that occur in the oral cavity. Several markers have been suggested as indicative of tumoral progression and aggressiveness, such as cyclooxygenase 2 (COX-2) and Ki67.

**Material and Methods:**

In this study, we have compared the expression of COX-2 and Ki67 in a series of amelanotic (n=7) and melanotic oral melanomas (n=22). The cases were selected from 4 pathology laboratories and submitted to the immunohistochemical (IHC) reactions. We analyzed the IHC staining based on a qualitative – using visual scores; and a computer-assisted method (quantitative) using scanned slides and software for digital analysis.

**Results:**

COX-2 was expressed in all oral melanomas; however, its intensity was significantly higher in the amelanotic ones (*P*<0.001). Similarly, a high Ki67-positivity index was observed in the amelanotic than melanotic ones (*P*<0.001).

**Conclusions:**

Based on these results, we suggest that amelanotic oral melanomas have marked pro-inflammatory and high-proliferative phenotype, justifying their more aggressive behavior compared with the melanotic ones.

** Key words:**Oral melanoma, amelanotic tumor, cyclooxygenase 2, Ki67.

## Introduction

Cutaneous and mucosal melanomas origin from cells derivate of the neural crest, the melanocytes (1). Clinically and microscopically, some tumors do not produce melanin, a brownish pigment that characterize these cells, and are named amelanotic tumors (2). In the cutaneous counterpart, the absence of melanin is considered a sign of aggressiveness (3). However, in the tumors of the oral cavity, the significance of this biological event is poorly understood.

Cyclooxygenase 2 (COX-2) is an enzyme involved in several inflammatory pathways. Its main function is the conversion of arachidonic acid to prostaglandin (4) COX-2 mediate several cellular functions, and in the context of the cancer, its higher expression is associated with modulation of angiogenesis, cellular migration, invasion and proliferation, as well as apoptotic resistance, events that favors tumor progression (5). In fact, COX-2 is associated with aggressiveness in non-small cell lung cancer (6), pancreatic ductal adenocarcinoma (7), hepatocellular carcinoma (8) and colorectal cancer (9).

Previous studies of our group have demonstrated that COX-2 expression is associated with angiogenesis in ameloblastoma (10) and cellular proliferation in cutaneous melanomas (11). However, to date, comparative studies of oral melanotic and amelanotic melanomas are not available in the English language literature. The aim of the present study was to evaluate the immunohistochemical expression of COX-2 and Ki67 in oral melanotic and amelanotic melanomas.

## Material and Methods

Formalin-fixed, paraffin-embedded tissue blocks and clinical information of 29 oral melanomas (7 amelanotic and 22 conventional melanotic) were obtained from the charts of four oral pathology laboratories from Latin America (Guatemala, Mexico and 2 from Brazil). Hematoxylin and eosin–stained slides were used for reviewing all diagnoses, and difficult cases were submitted to immunohistochemical analysis (HMB-45, S-100 and MelanA). To classify the melanomas selected in this study, we used a method proposed by Prasad *et al*. (12).

Accordingly previous protocols used in our laboratory, for immunohistochemical staining, 3-µm-thick sections were deparaffinized, rehydrated in graded ethanol solutions and after antigen retrieval with EDTA/Tris buffer (pH 9.0) in a pressure cooker; endogenous peroxidase activity was blocked with 20% H2O2 during 15 min. We used a method of 2-hours incubation with the primary antibodies: COX-2 (Clone: CX-294, dilution 1:100; Dako Corporation, Carpinteria, CA, USA) and Ki67 (Clone: MIB-1, dilution 1:100; Dako Corporation). Both diluted in BSA (bovine serum albumin). The secondary antibody conjugated with polymer dextran marked with peroxidase (Dako EnVision Labelled Polymer; Dako, Glostrup, Denmark) was applied for 1 hour. The reaction was developed with Permanent Red (Permanent Red Substrate System; Dako) and counterstained with Carazzi hematoxylin. Sections of oral squamous cell carcinoma were included in all reactions as positive control for both markers. Negative controls of reactions were performed by omitting the primary antibody. Only cytoplasmic staining was considered as positive for COX-2, and only nuclear reactivity was considered for Ki67. The quantification of the immunoreactivity was determined using a digital method previously validated by our group (10,13).

## Results

Amelanotic melanomas were more common in men (5 males, 2 females), with a mean age of 51.5 years. The most common location was gingiva (3 cases), followed by palate (2 cases), tongue (n=1) and lip (n=1). Five patients died from complications of the disease, 1 lost follow-up and 1 is disease-free. All but one patient was treated with surgery and complementary chemotherapy.

The conventional melanotic melanomas occurred predominantly in women (14 females, 8 males), with a mean age of 58 years. The locations were palate (n=14), gingiva (n=4) and other anatomical sites (n=4). Regarding treatment, 15 were treated with only surgery and 7 received complementary chemotherapy. Twelve patients died from complications of the tumor and 10 are alive.

**Figure 1:**
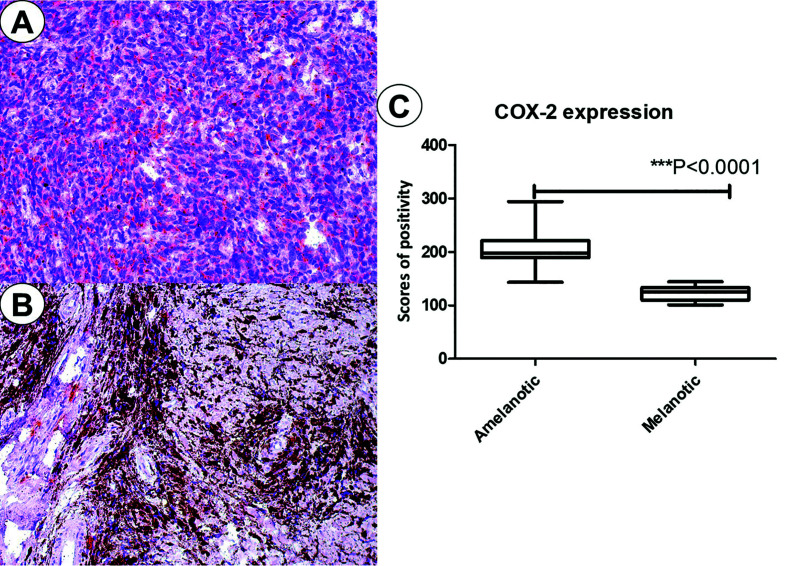
COX-2 expression in amelanotic (A) and melanotic melanomas (B). Graphical representation of the scores of immunopositivity.

COX-2 was expressed in the cytoplasm of tumor cells and occasionally in inflammatory cells. All melanomas were positive for COX-2 in different degrees. In the present series COX-2 expression had the highest positivity index for amelanotic (scores ranging from 143 to 294, mean 206), followed by melanotic melanomas (scores ranging from 101 to 144, mean 122.5). Representative photomicrographs are illustrated in the Fig. 1. The means were statistically different (t test, *P*<0.001).

**Figure 2:**
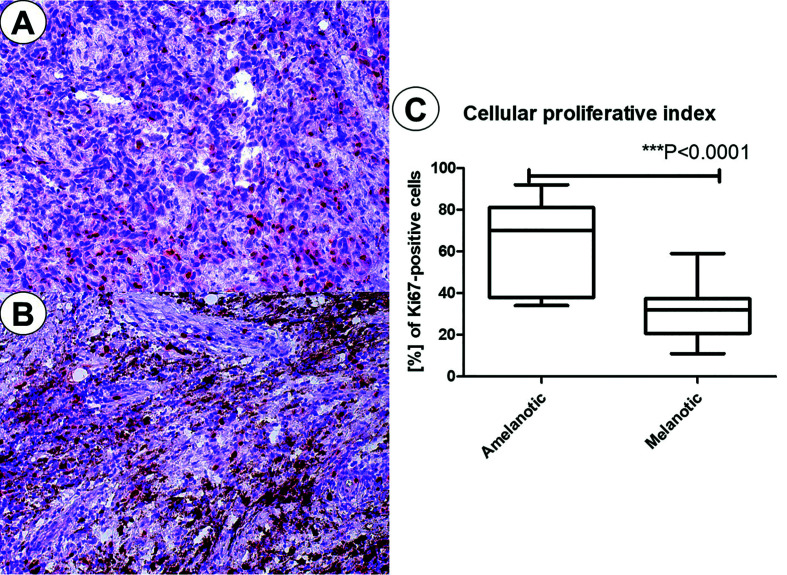
Ki67-nuclear expression in amelanotic (A) and melanotic melanomas (B). Graphical representation of the cellular proliferative index based on Ki67 expression.

Ki-67 presented a nuclear expression mainly in tumor cells. The index was digitally calculated and were higher for amelanotic (ranging from 34 to 92%, mean 64%) than melanotic melanomas (ranging from 11 to 59%, mean 30.8%). These results are described in the Fig. 2. The means were statistically different (t test, *P*<0.001).

## Discussion

To the best of our knowledge this is the first study to compare the expression of COX-2 and Ki67 in amelanotic versus conventional melanotic oral melanomas. In the present study we found that both studied markers demonstrated higher expression in amelanotic melanomas than melanotic ones. The main theory for these results, is that the amelanotic tumors represent a most undifferentiated neoplasm, and the presence of melanin may represent a more differentiated phenotype.

Other theory is that amelanotic tumors represent a de-differentiated conventional melanoma. In our opinion, it is very difficult to affirm which theory is more adequate. We only can confirm that amelanotic tumors really present a more aggressive behavior.

Tumor cell proliferation index is widely recognized to indicate the degree of aggressiveness of a tumor. In addition, Ki67-index has been used as prognostic factor for several tumors, including breast cancer (14) and mucosal melanomas (15). Although our data suggest a relationship between COX-2 expression and higher cell proliferation, other features must be considered including the idea that amelanotic tumors are more undifferentiated, have higher frequency of necrosis and mitosis than melanotic melanomas.

In fact, the higher expression of COX-2 in amelanotic corroborates with the hypothesis that these tumors have a poorer clinical prognosis, once COX-2 is involved in several pro-tumorigenic events, including angiogenesis (7), cell proliferation and migration (6) and association with a metastatic phenotype (11). Additional studies are necessary to determine the biological differences between amelanotic and melanotic oral melanomas.

In the cutaneous melanomas, the absence of melanin is an indicator of aggressiveness. Cheung *et al*. (3) have demonstrated that a higher mitotic index in amelanotic melanoma correlates with greater nuclear atypia and a worse prognosis. Similarly, our results demonstrated that oral amelanotic melanomas have a higher Ki67-index than the pigmented ones.

In summary, amelanotic melanomas had a higher COX-2 expression and higher cellular proliferative than melanotic ones. These data suggest that amelanotic melanomas differ from the melanotic subtype and more studies are necessary to better understand the role that the absence of melanin deposition may cause in the tumoral biology. It is important to keep in mind that, due to the rarity of these neoplasms, the data presented here must be interpreted with caution.
